# Tools for measuring technical skills during gynaecologic surgery: a scoping review

**DOI:** 10.1186/s12909-021-02790-w

**Published:** 2021-07-26

**Authors:** Louise Inkeri Hennings, Jette Led Sørensen, Jane Hybscmann, Jeanett Strandbygaard

**Affiliations:** 1 Department of Obstetrics and Gynaecology, Herlev Hospital, Herlev, Denmark; 2 Juliane Marie Centre for children, women and reproduction, Rigshospitalet, Copenhagen, Denmark; 3 Department of Clinical Medicine, University of Copenhagen, Copenhagen, Denmark; 4 Department of Obstetrics and Gynaecology, Rigshospitalet, Copenhagen, Denmark

**Keywords:** Assessment, Assessment tool, Gynaecology, Surgery

## Abstract

**Background:**

Standardised assessment is key to structured surgical training. Currently, there is no consensus on which surgical assessment tool to use in live gynaecologic surgery. The purpose of this review is to identify assessment tools measuring technical skills in gynaecologic surgery and evaluate the measurement characteristics of each tool.

**Method:**

We utilized the scoping review methodology and searched PubMed, Medline, Embase and Cochrane. Inclusion criteria were studies that analysed assessment tools in live gynaecologic surgery**.** Kane’s validity argument was applied to evaluate the assessment tools in the included studies.

**Results:**

Eight studies out of the 544 identified fulfilled the inclusion criteria. The assessment tools were categorised as global rating scales, global and procedure rating scales combined, procedure-specific rating scales or as a non-procedure-specific error assessment tool.

**Conclusion:**

This scoping review presents the current different tools for observational assessment of technical skills in intraoperative, gynaecologic surgery. This scoping review can serve as a guide for surgical educators who want to apply a scale or a specific tool in surgical assessment.

## Background

Surgical training has been shown to improve surgical skills, and several assessment tools have been validated in both live surgical settings and in a simulated environment [1]. Superior surgical performance is directly related to improved patient outcomes and **s**tandard-setting methods can define benchmarks and ensures content validity [2, 3]. The value of assessing a surgeon’s competencies is indisputable, but requires a trained assessor and an objective structured assessment tool [4]. Currently, there is no consensus on which scales to use in surgical assessment in gynaecologic surgery.

More than 20 years ago, Van der Vleuten explored and described five criteria (reliability, validity, impact on future behaviour, acceptability, and costs) to take into consideration when choosing an assessment method in the clinical setting [5]. They remain highly relevant, but especially reliability and validity must be thoroughly tested when applying an assessment tool.

Both task-specific and global rating tools are widely used in a variety of specialties [6]. The tools use various scoring systems, e.g. binary checklists or anchors, such as a Likert scale. In general surgery, a number of task-specific checklists exist, but recent reviews showed a lack of validity and reliability [7, 8].

Implementation of objective assessment in clinical practice is difficult due to challenges on many levels: lack of time, lack of resources, and often also lack of knowledge on how and when to use an assessment tool. To overcome these challenges it is important that the chosen assessment tool is acceptable, feasible, valid, well-described, and easy to apply in a surgical setting [5].

Our aim was to identify assessment tools measuring technical skills during live gynaecologic surgery and to evaluate the measurement characteristics of the tools used in a clinical setting in the operating room.

In this review, the term assessment tool refers to a specific tool that assesses specific surgical competencies, whereas the word scale refers to a widely applicable assessment tool or a component of a specific tool.

## Methods

We chose the scoping review methodology to characterise the quantity and quality of existing assessment scales [9]. Conducted in accordance with Arksey and O’Malley’s framework [10], the review was designed to cover all available literature on the topic, to summarise existing knowledge and to identify research gaps in the current literature. The underlying methodological framework comprised five consecutively linked stages (Table 1). Levac et al., has further developed Arksey and O’Malley’s approach in order to clarify and enhance the various stages and they recommend an optional sixth stage that involves consulting stakeholders [11]. The review is reported according to the Preferred Reporting Items for Systematic reviews and Meta-Analyses extension for Scoping Reviews (PRISMA-ScR) [12].

**Table 1:** Six consecutively linked stages of underlying methodological framework

Scoping review methodology based on Arksey and O’Malley	Arksey and O’Malley’s framework applied in scoping review
Review stage	
1. Identify research question	Studies relevant to research question; broad search strategy concerning assessment and gynaecologic surgery
2. Identify relevant studies	Information specialist assisted in the design and execution of a sensitive search strategy to include all relevant studies; electronic search of databases and reference lists; manual search of key journals.
3. Study selection	Broad inclusion criteria but studies performed in simulation settings or surgeries performed on animals were omitted, as the goal was assessment of gynaecologic surgery in a clinical setting.
4. Chart data	Overview noted author, assessment tool, study design, observation method and domains assessed during gynaecology surgery in a clinical setting.
5. Collate, summarise and report results	Aim of a scoping review is to present an overview of all available material on the research question. Data in the included studies were assessed an analysed with specific focus on the validity and feasibility of a given assessment tool. Each included study was critically reviewed in terms of strengths and limitations.
6. Consultation (optional)	Consulting with educational and gynaecological experts can provide further information and articles on the subject. Two of the authors are medical education and assessment experts and can contribute additional articles and expert knowledge.

### Search strategy

In accordance with stages one and two of Arksey and O’Malley’s methodology, we used a broad search strategy guided by an information specialist (PP). We identified keywords and created a search string (Appendix 1). On 1 of February 2020, four databases were searched covering 1989–2020: PubMed, Medline, Embase, and Cochrane. The search was updated on 8 January 2021.

We uploaded identified references into Covidence where two reviewers (LH, JH) screened the titles and abstracts and assessed the full texts in detail against the inclusion criteria [13]. The reference lists from the included studies were searched for additional studies.

### Inclusion/exclusion criteria

Inclusion of studies was based on the population (types of participants), concept, and context framework [14]. We included studies with the following types of participants: novices, junior doctors/ residents, and/or experts, whose technical skills were assessed (concept) during live gynaecologic surgery (context). Studies that assessed the competence of medical students were not included.

We included studies in English with empirical data that were published in peer-reviewed journals with no restriction of study design or publication year. We excluded studies assessing surgical performance on animals and studies evaluated in a simulation setting.

### Data synthesis

The measurement characteristics, i.e. validity, reliability, and validation context, were summarised for each type of assessment tool, see Table 2. Inspired by a recent systematic review by Hatala et al., we applied Kane’s validity argument, which comprises four inferences (Table 3) to evaluate the various assessment tools, see Table 4 [15, 16].

**Table 2:** Overview of studies identified for inclusion

Author and year	Assessment tool	Study design and assessment method	Domains assessed
**Hiemstra et al.** [17] 2011	**OSATS** **Objective Structured Assessment of Technical Skills**	*Observational study* Self-assessment and peri- and postoperative assessment by supervisor	*Generic scale* **1)** Respect for tissue **2)** Time and motion **3)** Knowledge and handling of instrument **4)** Flow of operation **5)** Use of assistants **6)** Knowledge of specific procedure
**Chen et al.** [18] 2010	**VSSI** Vaginal Surgical Skills Index	*Observational study* Assessment by supervisor and blinded reviewer of video recording	*Generic and procedure-specific scale* **1)** Initial inspection **2)** Incision **3)** Maintenance of visibility **4)** Use of assistants **5)** Knowledge of instruments **6)** Tissue and instrument handling **7)** Electro surgery **8)** Knot tying **9)** Haemostasis **10)** Procedure completion **11)** Time and motion **12)** Flow of operation and forward planning **13)** Knowledge of specific procedure
**Chou B et al.** [19] 2008	**HASC** Hopkins Assessment of Surgical Competency	*Observational study* Self-assessment and assessment by supervisor	*Generic and procedure-specific scale* *General surgical skills*: **1)** Knowledge/avoidance of potential complications, **2)** Respected tissue, **3)** Instrument Handling, **4)** Time and motion/moves not wasted, **5)** Bleeding controlled, **6)** Flow of operation *Specific surgical skills*: **1)** Knowledge of patient history/surgical indication, **2)** Knowledge of anatomy, **3)** Patient properly positioned on table/in stirrups, **4)** Proper placement of retractors, **5)** Proper assembly equipment, **6)** Proper positioning of lights
**Larsen CR et al.** [20] 2008	**OSALS** Objective Structured Assessment of Laparoscopic Salpingectomy	*Prospective cohort study* Blinded video assessment by two observers	*Generic and procedure-specific scale* *OSALS general skills* **1)** Economy of movement, **2)** Confidence of movement, **3)** Economy of time, **4)** Errors; respect for tissue, **5)** Flow of operation/operative technique *OSALS specific skills:* **1)** Presentation of anatomy, **2)** Use of diathermy, **3)** Dissection of fallopian tube, **4)** Care for ovary, ovarian artery and pelvic wall, **5)** Extraction of fallopian tube
**Peter J. Frederick et al.** [21] 2017	**RHAS** Robot Hysterectomy Assessment Score	*Observational study* Blinded video assessment by expert reviewers	*Procedure-specific scale* **1)** Handling of the round ligament, **2)** Developing the bladder flap, **3)** Isolating and securing the infundibulopelvic ligament (or utero-ovarian ligament if the ovaries were retained), **4)** Securing the uterine vessels, **5)** Performing the colpotomy and **6)** Closing the vaginal cuff
**Jeanne Goderstad et al.** [22] 2016	**CAT-LSH** Competence Assessment for Laparoscopic Supracervical Hysterectomy	*Prospective interobserver study* Blinded video assessment by expert reviewers	*Procedure-specific scale* **1)** Ligament mobilisation, **2)** Release of adnexa form uterus, **3)** Division of uterine vessels, **4)** Uterus amputation
**Savran et al.** [23] 2019	Feasible rating scale for formative and summative feedback	*Prospective cohort study* Blinded video assessment by two observers	*Procedure-specific scale* **1)** Division of fallopian tube and uteroovarian OR division of the infundibulopelvix ligament **2)** Dividing the round ligament **3)** Care for the ureter **4)** Opening the utero-vesicale peritoneum **5)** Identification and skeletonising **6)** Presentation and ligation of uterine arteries **7)** Opening of the vagina **8)** Suturing (catching the needle) **9)** Driving the needle through tissue, **10)** Placement and depth of sutures in the vaginal cuff, **11)** Suturing of the vagina and tying the knot
**Heinrich Husslein et al.** [24] 2015	**GERT** Generic Error Rating Tool	*Prospective observational study* Blinded video assessment by expert reviewers	*Error assessment - generic and procedure-specific scale* **1)** Abdominal access and removal of instruments or trocars, **2)** Use of retractors, **3)** Use of energy, **4)** Grasping and dissection, **5)** Cutting, transection and stapling, **6)** Clipping, **7)** Suturing, **8)** Use of suction, **9)** Other *Each generic task subdivided into four distinct error modes:* **(1)** Too much use of force or distance, **2)** Too little use of force or distance, **3)** Inadequate visualisation, **4)** Wrong orientation of instrument *Procedure subdivided into:* **1)** Insertion of trocars, **2)** Creation of bladder flap, **3)** Colpotomy **4)** Vault closure

## Results

We found eight articles: one global rating scale, three global and procedure rating tools combined, three procedure-specific rating tools and one non-procedure-specific error assessment tool.

**Fig. 1:**
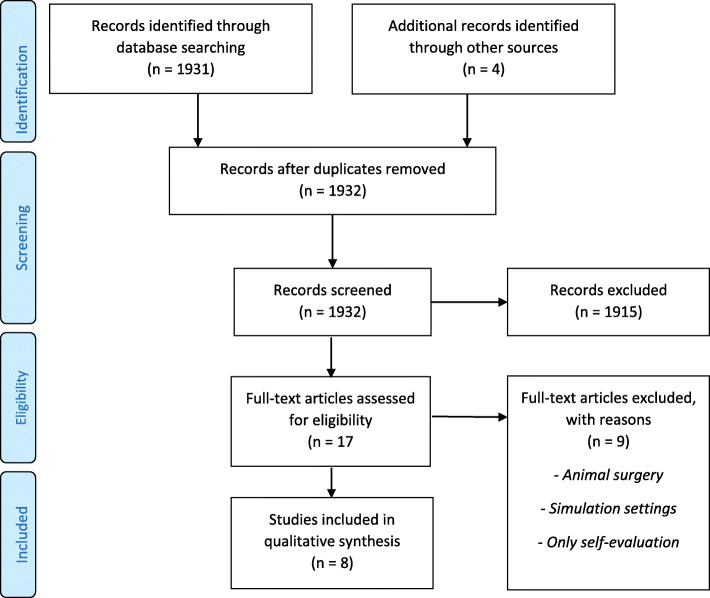
Flow diagram

Figure 1 contains a flowchart of the reference search, and Table 2 presents an overview of study characteristics for the eight articles that met our inclusion criteria. Table 4 present the studies evaluated by Kane´s validity argument.

**Table 3:** Kane’s validity argument

	Meaning
**Scoring**	**Observed performance on score or rating scale**
**Generalisation**	**Reflection of performance in test setting**
**Extrapolation**	**Use of scores to reflect on real-world performance**
**Implication/decision**	**Application of scores to make a decision or take action**

**Table 4:** Studies evaluated by Kane’s validity argument

Assessment tool	Scoring	Generalisation	Extrapolation
Objective Structured Assessment of technical Skills (OSATS) [17].	Comparison of OSATS scores over time.	Not reported	Construct validity was demonstrated as a significant rise in score with increasing caseload as 1.10 OSATS point per assessed procedure (*p =* 0.008, 95% CI 0.44–1.77)
Vaginal Surgical Skills Index (VSSI) [18].	Comparing GRS and VSSI. A visual analogue scale was added for overall performance.	Internal consistency for the VSSI and GRS = (Cronbach’s alpha (0.95–0.97)) Interrater reliability = 0.53 and intrarater reliability = 0.82	Construct validity was evaluated by measuring convergent validity using Pearson correlation coefficient (r) (VSSI = 0.64, *p* = 0.01, 95% CI 0.53–0.73) (GRS = 0.51, *p* = 0.001, 95% CI 0.40–0.61) and showed the ability to discriminate training levels by VSSI scores.
Hopkins Assessment of Surgical Competency (HASC) [19].	Surgeons rated on general surgical skills and case-specific surgical skills. No comparison.	Internal consistency reliability of the items using Cronbach’s alpha = 0.80 (*p* < 0.001)	Discriminative validity for inexperienced vs intermediate surgeons (*p <* 0.001)
Objective Structured Assessment of Laparoscopic Salpingectomy (OSA-LS) [20].	Surgeons rated by OSA-LS. No Comparison.	Interrater reliability =0.831. Intrarater reliability not reported.	Discriminative validity for inexperienced vs intermediate surgeon’s vs experienced surgeons (*p <* 0.03)
Robotic Hysterectomy Assessment Score (RHAS) [21].	Surgeons rated by expert viewers using RHAS. No Comparison,	Interrater reliability for total domain score = 0.600 (*p* < 0.001). Intrarater reliability not reported.	Discriminative validity for experts, advanced beginners and novice in all domains except vaginal cuff closure (*p* = 0.006).
Competence Assessment for Laparoscopic Supracervical Hysterectomy (CAT-LSH) [22].	Comparing GOALS and CAT-LSH	Interrater reliability = 0.75 Intrarater reliability not reported.	Discriminative validity for inexperienced vs intermediate (*p <* 0.001) and intermediate vs experts (*p <* 0.001) assessed by assistant surgeon. For blinded reviewers discriminative validity for inexperienced vs intermediate (*p* < 0.006) and intermediate vs experts (*p* < 0.011).
Feasible rating scale for formative and summative feedback [23].	Surgeons rated by expert viewers using 12-item procedure-specific checklist	Interrater reliability =0.996 for one rater and 0.0998 for two raters. Intrarater reliability not reported.	Discriminative validity for beginners and experienced surgeons (*p =* < 0.001)
GERT = Generic Error Rating Tool [24].	Comparing OSATS and GERT	Interrater reliability = > 0.95) Intrarater reliability = > 0.95)	Significant negative correlation between OSATS and GERT scores (rater 1: Spearman = − 0.76, (*p <* 0.001); rater 2 = − 0.88, (*p <* 0.001)

### Global rating scale (*N* = 1)

#### Objective structured assessment of technical skills (OSATS)

Hiemstra et al. [17] evaluated OSATS to establish learning curves and analyzed the scores of nine trainees over a three-month period. Nineteen types of procedures were identified among the 319 procedures assessed. The item “knowledge of instruments” and “instrument handling” from the original OSATS was merged and resulted in six assessment items.

The trainees and the supervising consultant were instructed to fill out an OSATS assessment sheet after a performed procedure. The consultant would discuss the result with the trainee and provide constructive feedback. Within the six OSATS items scores range from 6 to 30 points, and a score of 24 was the selected threshold for good surgical performance in the absence of benchmark criteria.

To prove construct validity, the authors hypothesise that surgical performance improves over time, with increasing procedure-specific experience [17]. They found that performance improved 1.10 OSATS points per assessed procedure (*p* = 0.008, 95% confidence interval (CI) 0.44–1.77) and that the learning curve for a specific procedure passed the threshold of 24 points at a caseload of five procedures. Furthermore, a performance plateau was reached after performing eight of the same procedures.

### Global and procedure-specific assessment tools combined (*N* = 3)

#### Vaginal surgical skills index (VSSI)

Chen et al. [18] Introduced Vaginal Surgical Skills Index (VSSI), a procedure-specific rating scale for evaluating surgeons while performing vaginal hysterectomies. They refined the original seven-item Global Rating Scale (GRS) to contain 13 items considered important for vaginal surgery.^1 2^

Twenty-seven trainees performed 76 surgeries in the study period. A supervisor assessed the trainee immediately after the surgery, using VSSI, GRS and a 100-mm visual analogue scale (VAS). VAS was included as an additional measure to furnish the assessor with a global impression of the trainees’ surgical skills. Construct validity was analysed by comparing VSSI and GRS scores, respectively. The procedure was videotaped, and to evaluate interrater reliability, a blinded reviewer assessed the performing surgeon using VSSI, GRS and VAS.

To evaluate intrarater reliability, the supervising surgeon watched and re-evaluated the video after 4 weeks. Internal consistency for VSSI and GRS was high (Cronbach’s alpha = 0.95–0-97). Inter- and intrarater reliability was ICC = 0.53 and ICC = 0.82, respectively.

#### Hopkins assessment of surgical competency (HASC)

Chou et al. developed and evaluated the Hopkins Assessment of Surgical Competency (HASC). The assessment form contains seven items from OSATS [25] and four items from an American Council on Resident Education in Obstetrics and Gynaecology toolbox. Another four items were included from an existing assessment tool at the author’s institution. After modifying the items by factor analysis, the results were two six-item scales; a General Surgical Skills and Case Specific Surgical Skills [19].

.Sixteen faculty physicians evaluated 16 trainees after performed surgery. The trainees also performed self-evaluation. The study analysed 362 cases, and the authors reported internal validity and reliability, demonstrated by high Cronbach’s alpha (>.80) and high Pearson correlation coefficients (> 0.80) for both scales. Discriminant validity was significant (*p <* 0.001) for both scales when comparing the performance of trainees in their second and fourth year of training.

#### Objective structured assessment of laparoscopic salpingectomy (OSALS)

Larsen et al. [20] developed and evaluated Objective Structured Assessment of Laparoscopic Salpingectomy (OSALS) a general rating scale and a case-specific scale, where three of the case-specific items are directly related to the procedure evaluated: laparoscopic salpingectomy. Two independent observers used the OSALS chart for assessment of 21 unedited video recordings of 21 laparoscopic salpingectomies, performed by 21 different surgeons grouped as either novices, intermediate or experts. The median score in each group proved construct validity and OSALS was able to discriminate between all groups (*p* < 0.03). The study found a wide performance range in the expert group and a narrow performance range in the novice group. Interrater reliability was reported acceptable.

### Task-specific assessment tools (*N* = 3)

#### Robotic hysterectomy assessment score (RHAS)

Frederick et al. [21] developed and evaluated Robotic Hysterectomy Assessment Score (RHAS). The assessment tool consists of six surgical domains on a five-point Likert scale, each domain subdivided into specific tasks with a possible maximum score of 80. Delphi methodology was used for content validation. The participating surgeons were grouped as experts, advanced beginners or novices. A blinded expert-reviewer evaluated fifty-two recorded procedures. Interrater reliability was acceptable and RHAS demonstrated construct validity, as it was able to differentiate between experts, advanced beginners and novices.

#### Competence assessment tool for laparoscopic Supracervical hysterectomy (CAT-LSH)

Goderstad et al. [22] developed Competence Assessment Tool for Laparoscopic Supracervical Hysterectomy (CAT-LSH), a procedure-specific rating tool for laparoscopic supracervical hysterectomy and compared it with Global Operative Assessment of Laparoscopic Skills (GOALS) [26], a general rating scale. GOALS has been validated for laparoscopic ventral hernia repair, laparoscopic appendectomy, laparoscopic inguinal hernia and laparoscopic and open cholecystectomy [7].

Three experts reached content validity by defining the main steps of the hysterectomy procedure. Each step evaluates the use of instruments, tissue handling and errors, with a maximum of 16 points assigned per step for a total possible score of 64. Twenty-one participants, grouped as either inexperienced, intermediate experienced or expert surgeons, performed 37 procedures eligible for assessment. The procedure was recorded, and the performing surgeon was assessed by both the operating assistant and by two blinded reviewers.

CAT-LSH proved significant discriminative validity as both the assistant surgeon and the blinded reviewers were able to discriminate between inexperienced, intermediate experienced and experts surgeons, respectively. The comparison, GOALS allowed blinded observers to differentiate between inexperienced and intermediate experienced surgeons, but not between intermediate experienced surgeons compared to expert surgeons (*p* = 0.085). When performed by the assistant surgeon, GOALS assessment differed significantly between the three groups. Acceptable interrater reliability was reported.

#### A feasible rating scale for formative and summative feedback

Savran et al. used Delphi methodology to develop the most recent procedure-specific rating scale, a feasible rating scale for formative and summative feedback [23]. The scale comprises 12 items evaluated on a five-point Likert scale. Messick’s framework was used to measure the validity evidence. Grouped as beginners (had performed < 10 procedures) or experienced surgeons (had performed > 200 procedures), 16 surgeons performed 16 laparoscopic hysterectomies. The procedure was video recorded and analysed by two blinded reviewers. Construct validity was demonstrated by significantly different mean scores of the two groups. High interrater reliability was found for both one and two raters.

### Non-procedure-specific error assessment (*N* = 1)

#### Generic error rating tool (GERT)

Husslein et al. [24] were the first to test a non-procedure-specific error assessment tool. The Generic Error Rating Tool (GERT) uses a Likert scale with nine anchors and is designed to analyse technical errors and resulting events during laparoscopy. GERT is based on the inverse relationship between surgeon and skill, i.e. more skilled surgeons make fewer errors.

Technical errors are defined as “the failure of planned actions to achieve their desired goal” and an event as “an action that may require additional measures to avoid an adverse outcome” [24]. The GERT technical error analysis comprises nine generic surgical tasks during which errors can occur. Each of these generic task groups is subdivided into four distinct error modes. To assess error distribution the procedures were divided into four sub-steps.

Two blinded reviewers analysed twenty video recordings of total laparoscopic hysterectomies, and correlation analyses were performed between GERT and OSATS. GERT scores were used to establish a measure of technical skills and to divide surgeons into two groups as either high or low performers. A significant negative correlation between OSATS and GERT scores were demonstrated. The total number of errors increased by increasing OSATS scores. Group comparison showed that high performers made significantly fewer technical errors than low performers.

By analysing the different operative sub-steps, the study detected procedures more prone to technical errors.

## Discussion

There is a need for robust validated tools across different measurement properties in order to aid surgical educators in selecting the appropriate tool for assessment. This scoping review identified eight technical assessment tools validated in live gynaecologic surgery. The studies, which have different validity strengths according to Kane’s framework, present a variety of challenges.

Currently, the most widely used and validated assessment scale is OSATS [25], which originally consisted of a task-specific checklist and a global rating scale. The global rating scale component have shown to have high reliability and validity and to be applicable at various trainee levels and for a variety of surgical procedures [27].

Hiemstra et al. [17] tested the OSATS intraoperatively to establish learning curves for each trainee by direct supervision or self-assessment. As expected, learning curves were established but the authors identified enormous variation in assessors’ OSATS scores, and the trainees reported a lack of objectivity in the assessment tool.

Lack of objectivity is an important limitation of the OSATS. According to Kane’s validity argument, it does not meet the extrapolation criteria, which implies that the scores cannot be used to reflect on real-world performance.^7^ Furthermore, the general rating scale seems to have a ceiling effect in terms of not being able to discriminate competencies for senior surgeons [28].

VSSI [18] was developed as a procedure-specific rating tool to assess surgeons while performing vaginal hysterectomies. Jelevsky et al. have further validated VSSI in a study where it was compared with GBS [29]**.**

Interestingly, the 13 items in VVSI are not procedure specific and can be applied to all laparoscopic surgery, e.g. general surgery.

This transfer of general competencies to a specific rating tool did not prove to be appropriate. Importantly, the authors focus on case mix, where a specific (patient) characteristic is known to potentially effect (surgical) outcome. A recent review on case-mix variables and predictors for outcomes of laparoscopic hysterectomy showed that body mass index, previous operations, adhesions and age were predominate case-mix characteristics [30]**.** This knowledge on case mix is important when choosing a surgical case for assessment.

Chou et al. modified an existing global rating scale by adding procedure specific items to develop HASC [19], which targets gynaecologic trainees and aims to evaluate all surgical competencies in gynaecologic surgery. This procedure-specific rating tool is applicable to all types of laparoscopic surgery. The generalizability and lack of a task-specific checklist makes HASC applicable to other gynaecologic programmes. To our knowledge, this applicability has not been demonstrated in other validated studies. The study was not blinded, only trainees were tested and data were collected for all types of surgical procedures, lowering the strength of the study.

The OSALS rating tools is incorporated in the Danish curriculum for assessment of OBGYN trainees [20, 31]. .It comprises five general and five task-specific items and was developed and validated in a blinded study [20]. Larsen et al. found a wide performance range in the expert group and a narrow performance range in the novice group. This could be explained by case mix and by the fact that the OSALS rating tools is difficult to use to assess expert. The study is limited by a small sample size.

A disadvantage of video evaluation is the time-consuming nature, but Larsen et al. underlined it as a strength for the objective assessment, an assertion supported by Langermann et al. [32] They argue that video recording of surgery enhances and supports surgical training and can be performed equally good by doctors with different experience [32, 33].

Six of the included studies used video recording and blinded observers when evaluating the surgeon’s performance [18, 21–24, 31]. All the studies found significant discriminative validity, demonstrating that the assessor can differentiate between novices, advanced beginners and experts. This indicates that video-recorded assessment is a good choice when validating an assessment tool, but as it is time-consuming, it may not be an obvious choice for implementation in daily clinical practice [34].

Even though the development of content validity for procedure-specific assessment tools requires the use of Delphi methodology [35] it was only done by Frederick et al. [21] They discussed the potentially confounding variable of the attending physician providing direct supervision and guidance when evaluating a novice surgeon. This may account for why novice surgeon’s scores did not differ more relative to their more experienced colleagues. Case mix may also explain this lack of difference in scores.

RHAS [21] demonstrated both construct and discriminative validity and appeared to be feasible. The assessed surgical skills can be applied to hysterectomies performed either laparoscopically or abdominally, as the basic steps in the procedure are identical.

The procedure-specific rating tool CAT-LSH was superior in terms of discriminative validity compared to the validated tool Global Operative Assessment of Laparoscopic Skills (GOALS) [26] used for laparoscopy. Goderstad et al. explain this by CAT-LSH being more detailed for each step of the procedure compared to GOALS. Frederick et al. who developed RHAS supports this finding [21]. The CAT-LSH study revealed rater bias, as that the operating assistant gave a higher total score than the blinded reviewer, both in terms of GOALS and CAT-LSH in all three groups. This phenomenon has also been demonstrated by Goff et al., who validated OSATS in a simulation setting [36].

Even though GOALS [26] is used as a comparison in the CAT-LSH study [22], the general rating scale has never been tested and validated in a live, gynaecologic surgical setting. Interestingly, that is also the case for the most widely used global assessment scale, OSATS. GOALS was developed and validated to assess and intraoperative laparoscopic skills in general surgery [26] and OSATS was originally validated for general surgery in a simulation setting [25]. A comprehensive study by Hatala et al. [16] thoroughly analysed the validity evidence for OSATS in a simulating setting and a recent study has developed an H-OSATS, an objective scale specific for the assessment of technical skills for laparoscopic hysterectomy [37]. It showed feasibility and validity but was only tested in a simulation setting. The global rating scale must still be validated in intraoperative gynaecologic surgery before it can be used as a validated comparison.

Husslein et al. examined GERT [24] which was able to significantly discriminate between low and high performers by analysing errors. The study identified procedures more prone to technical errors, which is important knowledge when determining the focus of a procedure-specific assessment tool and how detailed each procedural step should be evaluated. The study is limited by a small sample size.

A systematic review by Ahmed et al. [38] concluded that a combination of global and task-specific assessment tools appears to be the most comprehensive solution for observational assessment of technical skills. This is supported by findings in the RHAS, CAT-LSH and OSALS. These scales all consist of a general and procedure-specific checklist and have been validated in studies with relatively strong methodology. It has been shown in a simulation setting that evaluation of a clinical competence solely using a procedure-specific checklist, does not preclude incompetence in terms of technical ability and safety [39]. Identifying safety issues requires the inclusion of assessment using a global rating scale. By adding GERT the operative substeps prone to errors can be identified.

Savran et al. [23] asserted that their assessment tool met the criteria for summative assessment, using the contrasting group method to set a pass/fail score. Similar to most studies in our scoping review, the authors grouped surgeons according to surgical load, and experienced or expert surgeons were defined according to the number of cases performed. This is not an objective measure of competency, just as a pre-set standard must exist in order to establish summative assessment [4].

Focused on formative feedback, high-stakes assessment and programme evaluation, Hatala et al. [16] used Kane’s framework to evaluate OSATS and found reasonable evidence in terms of scoring and extrapolation for formative and high-stakes assessment. For programme assessment, there was validity evidence for generalisation and extrapolation but a complete lack of evidence regarding implications and decisions based on OSATS scores. This calls for more research.

The majority of surgical assessment scales are validated in a simulation setting, where the cognitive and communicative mechanisms of action are less complex compared to the operating room setting. An assessment scale validated in a simulated setting can hence not be transferred to live surgery, especially not in terms of summative assessment.

### Limitations

This scoping review has some limitations. First, it is limited by only including studies in English. We included peer review published literature that did include grey literature. Also, it is a limitation that the majority of the included studies are small, only conducted once and without a comparison group. There is a need for larger, randomised studies to evaluate their validity before they are enroled in gynaecological curricula or used for summative assessment.

## Conclusion

We identified eight tools measuring technical skills during gynaecologic surgery, all of which depend on the user context, with varying validity frameworks. A combination of global and task-specific assessment tools with a focus on operative sub-steps prone to errors may by an approach when assessing surgical competencies in gynaecology.

This scoping review can serve as a guide for surgical educators who wish to evaluate surgical assessment.

## Data Availability

Not applicable.

## Appendix 1

Search strings *(((((“Gynecologic Surgical Procedures”[Mesh]) OR “Gynecology”[Majr])) AND ((“Surgical Procedures, Operative”[Mesh]) OR “General Surgery”[Majr])) AND (((tool) OR instrument) OR scale)) AND assessment*.

We searched four databases (PubMed, Medline, Embase and Cochrane) using the free text terms gynaecology OR general surgery AND tool OR instrument OR Scale OR instrument. We combined these with non-Medical Subject Headings (MeSH) words “gynaecologic surgical procedures” and “operative surgical procedures”. The Cochrane database was searched for reviews on the subject; none were found. No conference abstracts were found.

## Abbreviations

PRISMA-ScR: Preferred Reporting Items for Systematic reviews and Meta-Analyses extension for Scoping Reviews
CI: Confidence interval
ICC: Intraclass correlation coefficient
OSATS: Objective Structured Assessment of Technical Skills
VSSI: Vaginal Surgical Skills Index
GRS: Global Rating Scale
HASC: Hopkins Assessment of Surgical Competency
OSALS: Objective Structured Assessment of Laparoscopic Salpingectomy
RHAS: Robotic Hysterectomy Assessment Score
CAT-LSH: Competence Assessment Tool for Laparoscopic Supracervical Hysterectomy
GOALS: Global Operative Assessment of Laparoscopic Skills
GERT: Generic Error Rating Tool
